# Response to recombinant human granulocyte colony-stimulating factor in reticular dysgenesis

**DOI:** 10.70962/jhi.20250232

**Published:** 2026-04-06

**Authors:** Manabu Wakamatsu, Yohko Kitagawa, Yusuke Tsumura, Yusuke Okuno, Yoshitaka Sato, Yoshiro Kamachi, Ryo Maemura, Masayuki Imaya, Ayako Yamamori, Kotaro Narita, Shinsuke Kataoka, Atsushi Narita, Nobuhiro Nishio, Seiji Kojima, Yoshiyuki Takahashi, Megumu K. Saito, Hideki Muramatsu

**Affiliations:** 1 Department of Pediatrics, Nagoya University Graduate School of Medicine, Nagoya, Japan; 2 Department of Clinical Application, Center for iPS Cell Research and Application (CiRA), Kyoto, Japan; 3 Department of Virology, Nagoya City University Graduate School of Medical Sciences, Nagoya, Japan; 4 Department of Virology, Nagoya University Graduate School of Medicine, Nagoya, Japan

## Abstract

Reticular dysgenesis (RD) is characterized by severe combined immunodeficiency and agranulocytosis with hematopoietic stem cell transplantation (HSCT) being the only curative therapy. Severe neutropenia in RD is typically unresponsive to recombinant human granulocyte colony-stimulating factor (rhG-CSF). To delineate lineage-specific transcriptional responses and cellular heterogeneity induced by rhG-CSF, we integrated single-cell RNA sequencing from seven samples, including two RD patients (with and without rhG-CSF) and three pediatric controls. We identified a moderate increase in hematopoietic stem and progenitor cells (HSPCs) and common myeloid progenitor/granulocyte–monocyte progenitor fractions following rhG-CSF. Notably, B cell fractions increased after rhG-CSF, accompanied by enhanced maturation from common lymphoid progenitors to precursor B cells. In parallel, genes involved in B cell differentiation were markedly upregulated compared with untreated cells. It also modulated the HSPC compartment by markedly suppressing interferon-γ (IFNγ) signaling pathways. RhG-CSF administration before HSCT may benefit patients with RD by modestly increasing neutrophils and supporting infection control, while suppressing IFNγ signaling in HSPCs and potentially promoting B cell differentiation.

## Teaser

Wakamatsu et al. assess transcriptional and cellular responses to rhG-CSF in reticular dysgenesis using single-cell RNA sequencing. The study demonstrates that rhG-CSF modestly expands hematopoietic progenitors, suppresses IFNγ signaling, and promotes B cell differentiation, suggesting potential benefits for infection control and immune development before transplantation.

## Graphical Abstract

**Figure:**
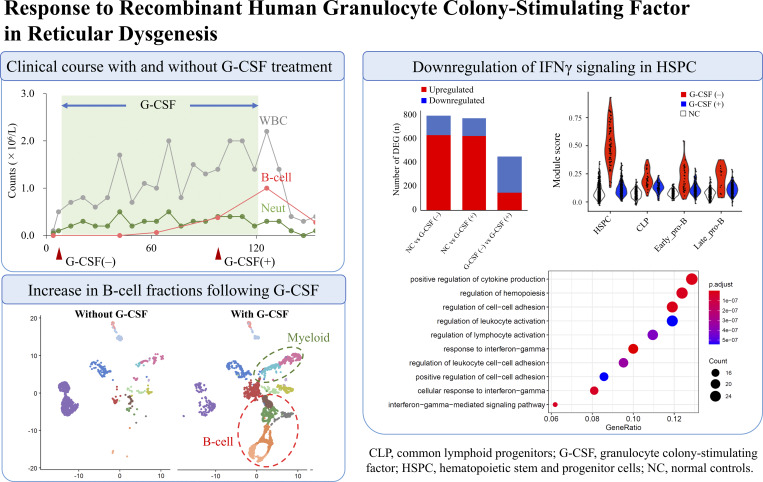

## Introduction

Severe combined immunodeficiency (SCID) encompasses a group of disorders characterized by abnormal T and B lymphocyte development and function, typically leading to death within the first year of life (1, 2). Reticular dysgenesis (RD) (Mendelian Inheritance in Man #267500) is an extremely rare and most severe form of SCID due to impaired intracellular adenosine triphosphate transport in the mitochondrial intermembrane space caused by adenylate kinase 2 (AK2) deficiency (3). It is characterized by profound neutropenia and lymphopenia, resulting in a high susceptibility to not only viral and fungal infections but also life-threatening bacterial infections, with bilateral or unilateral sensorineural hearing loss. RD is fatal within days to months without rapid diagnosis and curative hematopoietic stem cell transplantation (HSCT) due to overwhelming bacterial infection (4). Recombinant human granulocyte colony-stimulating factor (rhG-CSF) is a synthetic form of G-CSF that promotes the proliferation, survival, and maturation of myeloid progenitor cells, mimicking the physiological functions of endogenous G-CSF (5, 6). The standard clinical criteria for RD include severe neutropenia typically unresponsive to rhG-CSF (7). However, the pathological mechanism underlying the rhG-CSF insensitivity of *AK2* gene variants remains to be elucidated.

Recent studies have evaluated the role of inflammatory cytokines, including interferon γ (IFNγ), in the regulation of hematopoiesis (8). While IFNγ plays a critical role in immune responses such as macrophage activation and antigen presentation, its excessive production can suppress hematopoietic progenitor proliferation and contribute to cytopenic disorders, including hemophagocytic lymphohistiocytosis (HLH) and aplastic anemia (AA) (9). This cytokine-induced suppression of hematopoiesis is particularly relevant in the context of severe infections and inflammatory conditions, as it regulates both the proliferation and apoptosis of hematopoietic progenitor cells (10).

Here, we report two cases of RD who were administered rhG-CSF prior to HSCT. Both cases maintained neutrophil counts and showed recovery of the B cell fraction following rhG-CSF administration. We performed single-cell RNA sequencing (scRNA-seq) to comprehensively assess lineage-specific transcriptional responses and cellular heterogeneity between samples with and without rhG-CSF treatment.

## Results

### Clinical course of two patients with RD

**Figure 1.**
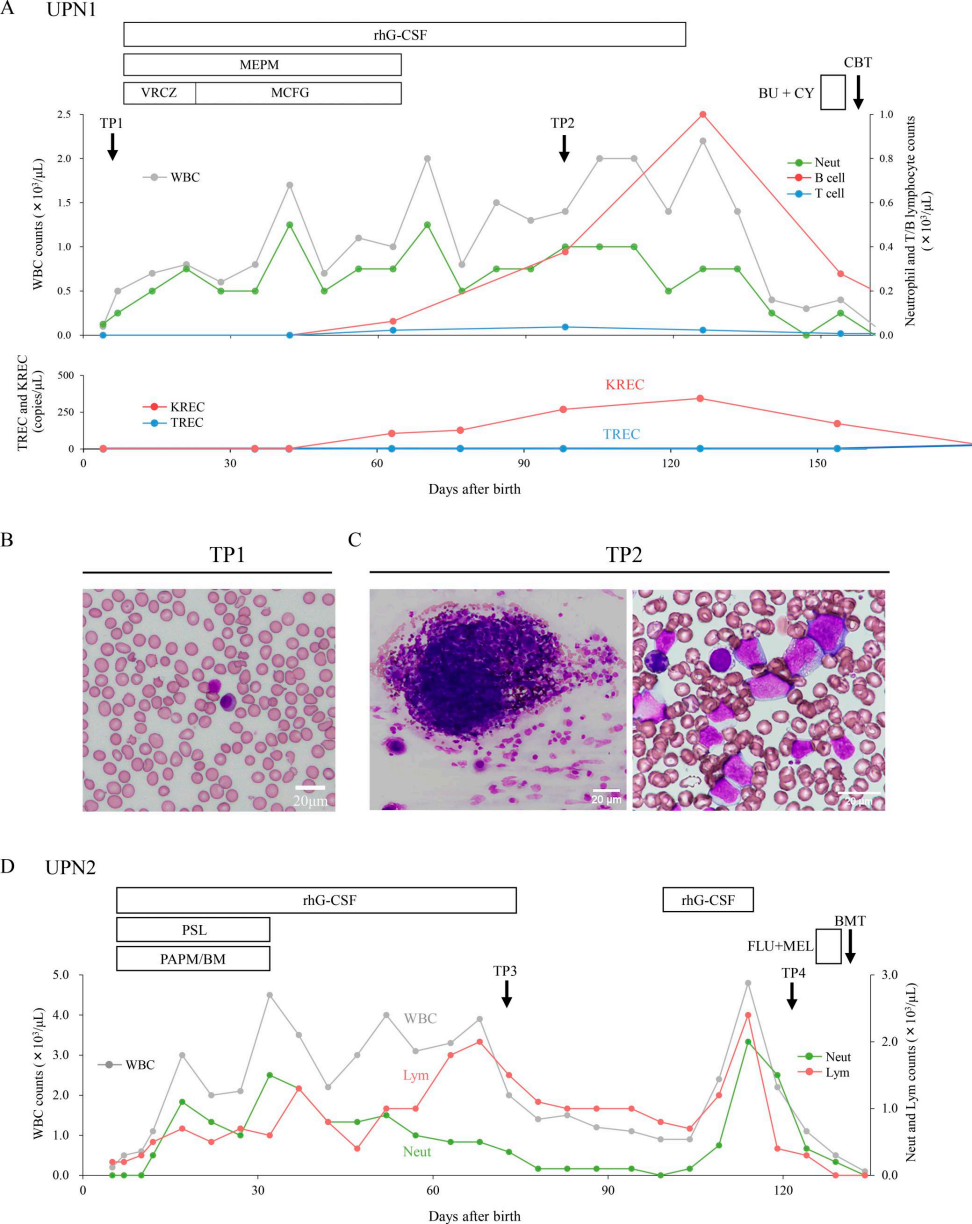
**Clinical presentations of two patients with RD with and without rhG-CSF administration before HSCT. (A)** Clinical presentation of UPN1 with RD. BMA was performed without rhG-CSF administration on postnatal day 8 (TP1) and with its administration on postnatal day 97 (TP2). On 35 days of age, KREC values and B lymphocyte counts were moderately increased. **(B and C)** TP1 (B) and TP2 (C) of the BM smear. Hematopoietic progenitor cells, particularly within the myeloid lineage, increased following rhG-CSF administration. **(D)** Clinical presentation of UPN2 with RD. BMA was performed on postnatal day 75 with rhG-CSF administration (TP3) and without it before HSCT (TP4). BMA, bone marrow aspiration; BM, betamipron; BU, busulfan; CY, cyclophosphamide; CBT, cord blood transplantation; FLU, fludarabine; HSCT, hematopoietic stem cell transplantation; KREC, kappa-deleting recombination excision circle; MCFG, micafungin; MEL, melphalan; MEMP, meropenem; PAPM, panipenem; PSL, prednisolone; RD, reticular dysgenesis; rhG-CSF, recombinant human granulocyte colony-stimulating factor; TP, time point; UPN, unique patient number; VRCZ, voriconazole; WBC, white blood cell.

Unique patient number (UPN)1 showed urgent abnormal T cell receptor excision circle (TREC, 0 copy/μl) and κ-deleting recombination excision circle (KREC, 0 copy/μl) values at 4 days old in the newborn screening (NBS) program for inborn errors of immunity in Aichi, Japan (Fig. 1 A) (11). The results became available on day 6, and the screening center immediately contacted the clinical facility. Coincidentally, on the same day (day 6), the patient developed a fever and was admitted to another hospital, where empirical antibiotic therapy was initiated. She presented leukopenia, absence of neutrophils, and profound hearing loss. The extremely low CD3^+^ T cell (0.05 × 10^9^/L), CD4^+^ T cell (0.02 × 10^9^/L), CD4^+^CD45RA^+^ T cell (0.01 × 10^9^/L), CD19^+^ B cell (<0.01 × 10^9^/L), and CD16^+^CD56^+^ natural killer (NK) cell (0.01 × 10^9^/L) levels were consistent with a T^−^B^−^NK^−^ SCID phenotype (Table 1). An extra-hypoplastic marrow was observed with a failure of myeloid maturation and a developmental arrest at the promyelocytic stage (Fig. 1 B). Next-generation sequencing analysis identified compound-heterozygous *AK2* variants (c.498+1G>A splice site variant and c.409C>T, p.Arg137Ter) that are diagnostic of RD. To prevent life-threatening bacterial and/or fungal infections, she was treated with intravenous rhG-CSF administration, antibiotics, antifungal agents, and immunoglobulin replacement therapy. The number of neutrophils and B lymphocytes was not detected before rhG-CSF administration. Following rhG-CSF administration, the white blood cell, neutrophil, and CD19^+^ B lymphocyte counts were increased by 1.0–2.0 × 10^9^/L, 0.3–0.5 × 10^9^/L, and up to 1.0 × 10^9^/L, respectively, whereas the CD3^+^ T lymphocyte count remained extremely low at <0.1 × 10^9^/L (Fig. 1 A). Furthermore, KREC levels, initially undetectable (0 copies/μl) without rhG-CSF, increased to a peak of 343 copies/μl during the 129-day course of treatment with rhG-CSF. After discontinuation of rhG-CSF shortly before HSCT, KREC levels dropped back down within a few days. Bone marrow (BM) smears revealed a severely hypoplastic marrow without rhG-CSF administration (TP1), which subsequently shifted to a hyperplastic marrow after rhG-CSF treatment (TP2), showing progression of myeloid maturation with proliferation of dysplastic myeloid lineage cells, without an accompanying increase in blast cells (Fig. 1, B and C). G-banding analysis performed at TP2 showed a normal karyotype. We performed cord blood transplantation (CBT) following a conditioning regimen of busulfan and cyclophosphamide at 5 mo old. 6 mo after CBT, sustained complete donor chimerism of granulocyte and T and B lymphocyte subsets was observed following neutrophil engraftment.

**Table 1.** Clinical characteristics in two patients with RD

​	UPN1	UPN2
Gender	Female	Female
Body weight (g)	3,086	3,068
Gestation (week)	38	36
TREC (copies/μl)	0	<10 copies/µg DNA
KREC (copies/μl)	0	NA
ANC (×10^9^/L)	0.04	0.01
ALC (×10^9^/L)	0.06	0.14
CD3^+^ (×10^9^/L)	0.05	0.01
CD4^+^CD45RA^+^ (×10^9^/L)	0.01	0.01
CD19^+^ (×10^9^/L)	0.01	0.04
CD16^+^CD56^+^ (×10^9^/L)	0.01	0.03
Physical findings	Athymia, ventricular septal defect, sensorineural deafness	Thymic hypoplasia, sensorineural deafness
Gene variants	*AK2*, NM_001625.4: c.498+1G>A and c.409C>T, p.Arg137Ter	*AK2*, NM_001625.4: c.308G>A, p.Arg103Gln and c.409C>T, p.Arg137Ter
Total period of rhG-CSF administration before HSCT (days)	129	78
Dose of rhG-CSF	40 µg/total	75 µg/total
Conditioning regimen	BU + CY	FLU + MEL
Graft source	UR-CBT	R-BMT

UPN, unique patient number; ANC, absolute neutrophil count; ALC, absolute lymphocyte count; TREC, T cell receptor excision circle; KREC, κ-deleting recombination excision circle; HSCT, hematopoietic stem cell transplantation; G-CSF, granulocyte colony-stimulating factor; BU, busulfan; CY, cyclophosphamide; MEL, melphalan; UR-CBT, unrelated cord blood transplantation; R-BMT, related bone marrow transplantation; NA, not assessed.

UPN2 initially presented with mildly decreased oxygen saturation after birth. A blood test showed leukopenia (2.90 × 10^9^/L), which further declined on day 2 to 1.50 × 10^9^/L, with neutrophils at 0.12 × 10^9^/L and lymphocytes at 1.02 × 10^9^/L. C-reactive protein was also elevated to 2.3 mg/dl. rhG-CSF and empirical antibiotic therapy were administered, and she was referred to our department and hospitalized for further evaluation. Flow cytometric analysis at the time of admission revealed an extremely low number of CD4^+^CD45RA^+^ naïve T cells (0.01 × 10^9^/L), along with a marked decrease in total peripheral blood (PB) lymphocyte counts and poor mitogen responses, fulfilling the diagnostic criteria for SCID (Table 1). Approximately 14% of the PB lymphocytes were CD8^+^ cells, predominantly expressing HLA-DR and CD45RO. CD8^+^, CD19^+^, and CD56^+^ lymphocytes were isolated from the patient’s BM, and short tandem repeat analysis revealed that the CD8^+^ cells were of maternal origin, while the CD19^+^ and CD56^+^ cells were derived from the patient. Genetic analysis subsequently identified compound-heterozygous variants in the *AK2* gene (c.308G>A, p.Arg103Gln and c.409C>T, p.Arg137Ter), confirming the diagnosis of RD. Consequently, rhG-CSF, antibiotics, antifungal drugs, and immunoglobulin replacement therapy were initiated. After the initiation of rhG-CSF, a moderate response to rhG-CSF was observed as evidenced by an increased neutrophil count (0.5–1.0 × 10^9^/L), which improved severe bacterial infection (Fig. 1 D). At 4 mo of age, bone marrow transplantation was performed from HLA-matched sibling donor using a nonmyeloablative conditioning with fludarabine and melphalan, and successful immune reconstitution was achieved after HSCT without any transplant-related complications.

### Altered gene expressions between rhG-CSF(+) and rhG-CSF(−) samples in patients with RD

**Figure 2.**
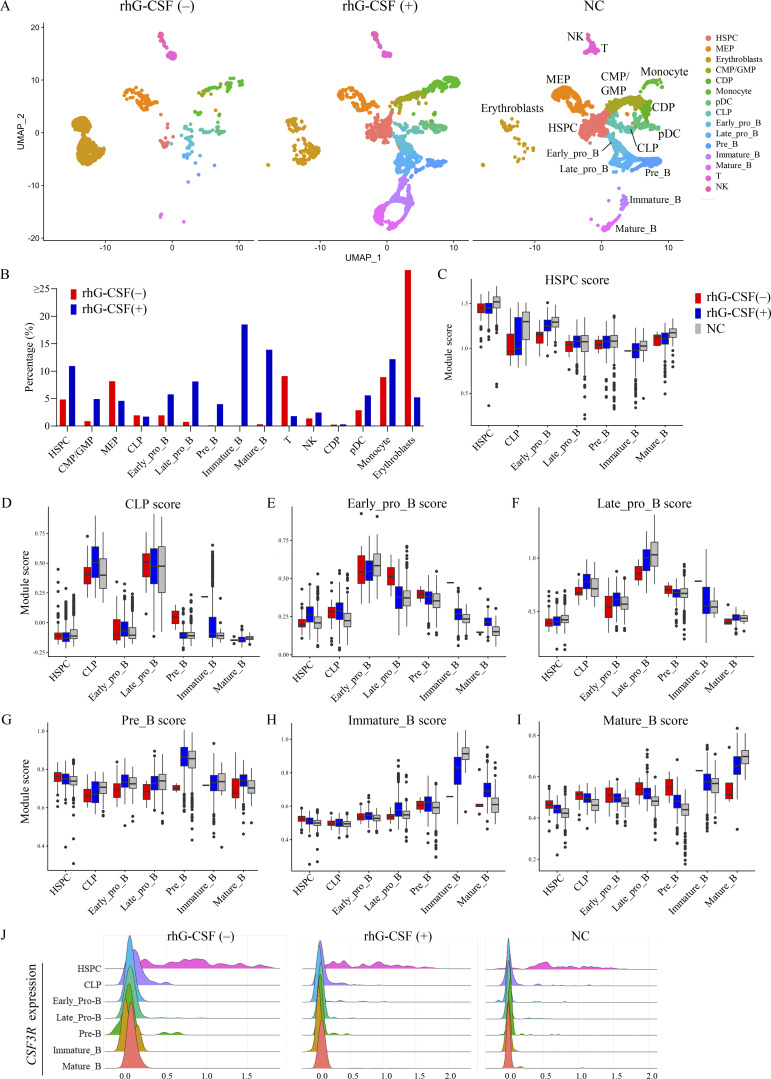
**Gene expression alterations with and without rhG-CSF administration using scRNA-seq. (A)** Cell-type annotation based on integrated scRNA-seq analysis of UPN1 and UPN2 with and without rhG-CSF administration, and NC samples. **(B)** Proportion of identified cell types, divided into three groups: rhG-CSF (−), rhG-CSF (+), and NC groups. **(C–I)** Module score for with and without rhG-CSF groups based on the significantly DEG sets for each cluster in NCs: HSPC score (C), CLP (D), early pro-B (E), late pro-B (F), pre-B (G), immature B (H), and mature B (I). Boxplots show module scores. Box = 25–75th percentile, line = median, whiskers = 1.5× interquartile range, points = outliers. **(J)** Comparison of *CSF3R* (G-CSF receptor) gene expression levels in HSPC and B cell fractions across three groups. CDP, common dendritic cell progenitor; CMP/GMP, common myeloid progenitors/granulocyte–monocyte progenitors; CLP, common lymphoid progenitors; CSF3R, colony-stimulating factor 3 receptor; HSPC, hematopoietic stem and progenitor cell; MEP, megakaryocyte–erythroid progenitor; NC, normal control; pDC, plasmacytoid dendritic cell; rhG-CSF, recombinant human granulocyte colony-stimulating factor; scRNA-seq, single-cell RNA-seq; UPN, unique patient number.

**Figure S1.**
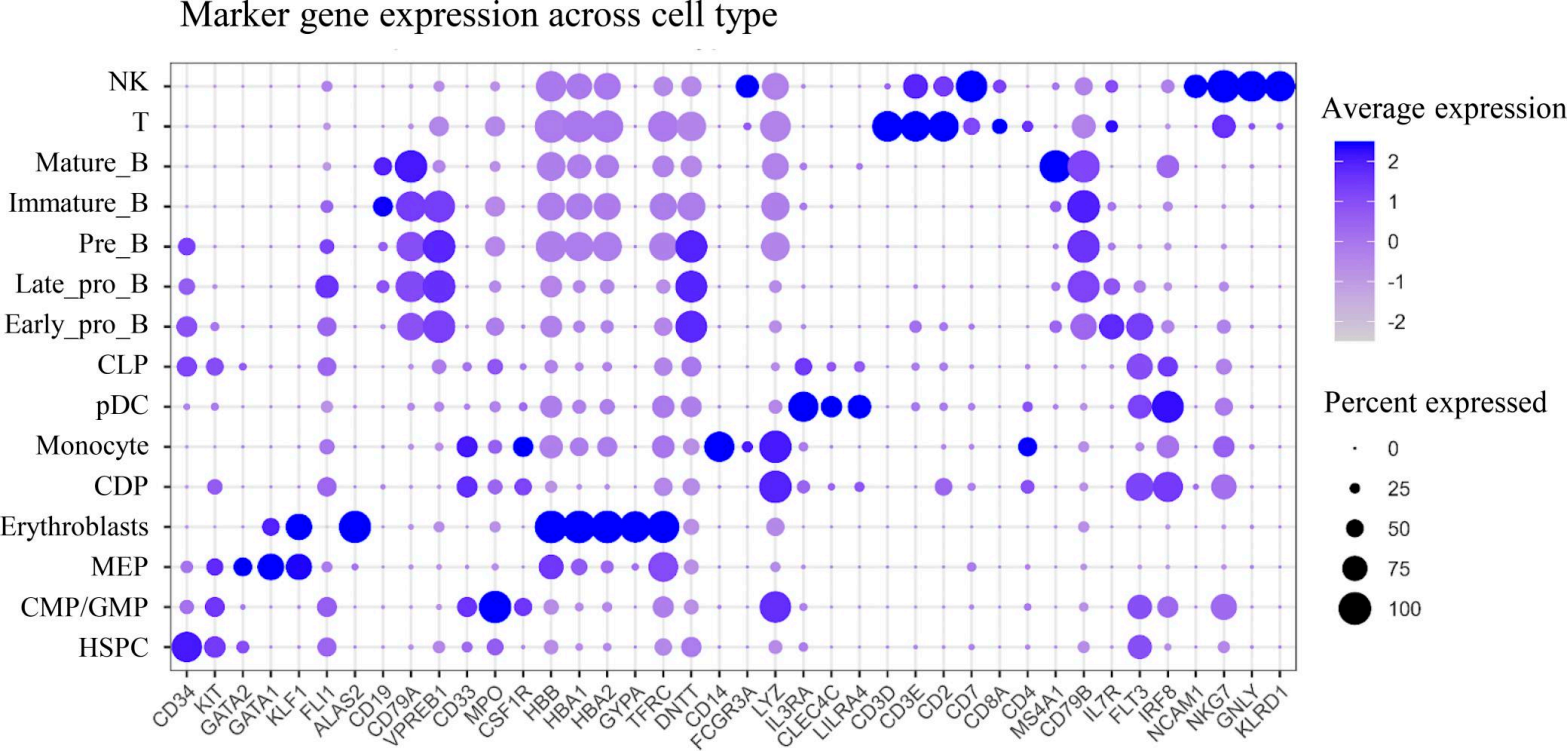
**Expression of representative marker genes across hematopoietic cell types.** Hematopoietic cell populations were defined and annotated using established marker gene combinations. The observed cell type–specific expression patterns of these canonical markers across annotated clusters are consistent with the defined annotation. CDP, common dendritic cell progenitors; CLP, common lymphoid progenitors; CMP, common myeloid progenitors; GMP, granulocyte–monocyte progenitors; HSPC, hematopoietic stem and progenitor cell; MEP, megakaryocyte-erythroid progenitors; pDC, plasmacytoid dendritic cell.

We performed scRNA-seq on four bone marrow mononuclear cell (BMNC) samples from two patients with RD, both with and without rhG-CSF administration, and integrated these data with scRNA-seq profiles from three BMNC samples derived from pediatric healthy controls (normal controls, NCs). We identified 15 distinct clusters using reference-based cell-type annotation, and compared the proportions of each cell type across the three groups: without rhG-CSF, with rhG-CSF, and NC (Fig. 2 A and Fig. S1). Indeed, hematopoietic stem and progenitor cells (HSPCs) and common myeloid progenitors/granulocyte–macrophage progenitors increased with rhG-CSF administration (Fig. 2 B). Notably, in the rhG-CSF(+) group, the proportion of B cell fractions beyond the early pro-B stage markedly increased, with sequential maturation observed from common lymphoid progenitors (CLP) through early/late pro-B, pre-B, immature B, and mature B stages (Fig. 2 B).

To assess how rhG-CSF induces changes in cellular signaling and cytokine profiles, we analyzed gene expression alterations associated with the increased B cell fractions. For this purpose, cell type–specific scores were established on the basis of the top 200 significantly differentially expressed genes (DEGs) identified for each cell type in the NC group, and these scores were subsequently employed to delineate the characteristics of each cell type in the presence or absence of rhG-CSF administration. Cell type–specific module scores across B cell maturation stages, including CLP, early/late pro-B, pre-B, immature B, and mature B cells, were calculated based on gene sets preferentially expressed in the NC group. Compared with the rhG-CSF(−) group, module scores were increased in the rhG-CSF(+) group from the late pro-B stage onward, indicating enhanced B cell maturation at more differentiated stages (Fig. 2, C–I). To further investigate the effects of rhG-CSF, we assessed the gene expression of *CSF3R*, the receptor for G-CSF, in each cell fraction with and without rhG-CSF administration. Without rhG-CSF, *CSF3R* was expressed predominantly in the HSPC fraction, with expression gradually declining as differentiation progressed (Fig. 2 J), indicating that HSPC in RD patients may retain the capacity to respond to rhG-CSF.

**Figure 3.**
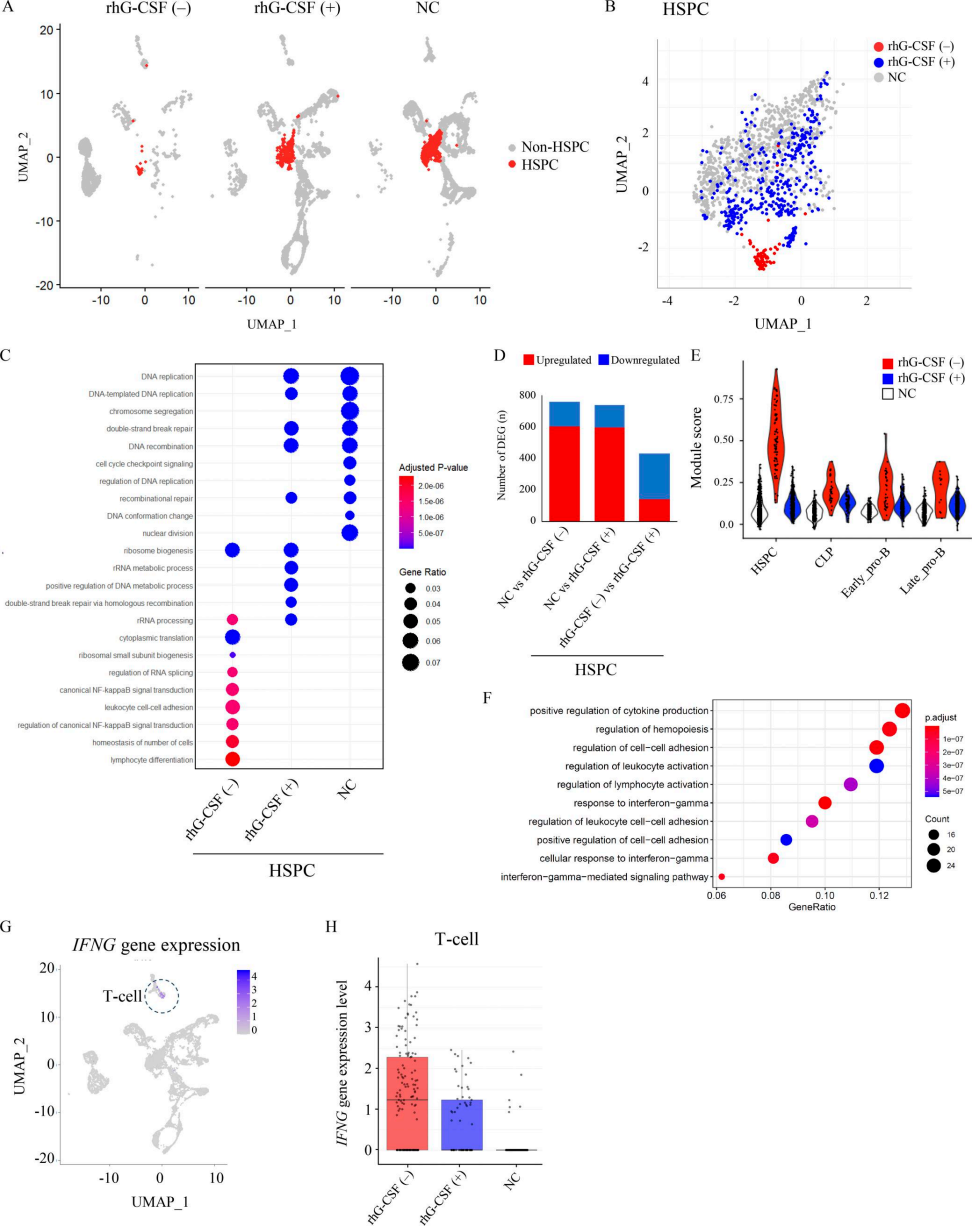
**Downregulation of IFNγ signaling in the HSPC fraction in rhG-CSF(+) samples. (A)** HSPC fraction in the with rhG-CSF, without rhG-CSF, and NC groups. **(B)** Principal component analysis of gene expression profiles in the HSPC fraction across three groups. **(C)** Enrichment analysis of genes with significantly altered expression in the HSPC fraction of each group. The HSPC fraction without rhG-CSF administration demonstrated upregulation of the NF-κB pathway and lymphocyte-related pathways. **(D)** Number of up- and downregulated genes with significant expression alteration between the three groups. **(E)** Module scores based on gene sets (*n* = 244) with significantly decreased expression with rhG-CSF administration compared to without it. **(F)** Gene set enrichment analysis of these 244 genes, performed specifically on the HSPC cluster, showing a marked reduction in IFNγ-related signaling pathways. **(G and H)** Gene expression plot of *IFNG*. **(H)** Comparison of *IFNG* gene expression in the T cell fraction with and without rhG-CSF administration. HSPC, hematopoietic stem and progenitor cells; IFNγ, interferon γ; NC, normal controls; NF-κB, nuclear factor κ-light-chain-enhancer of activated B cells; rhG-CSF, recombinant human granulocyte colony-stimulating factor.

**Figure S2.**
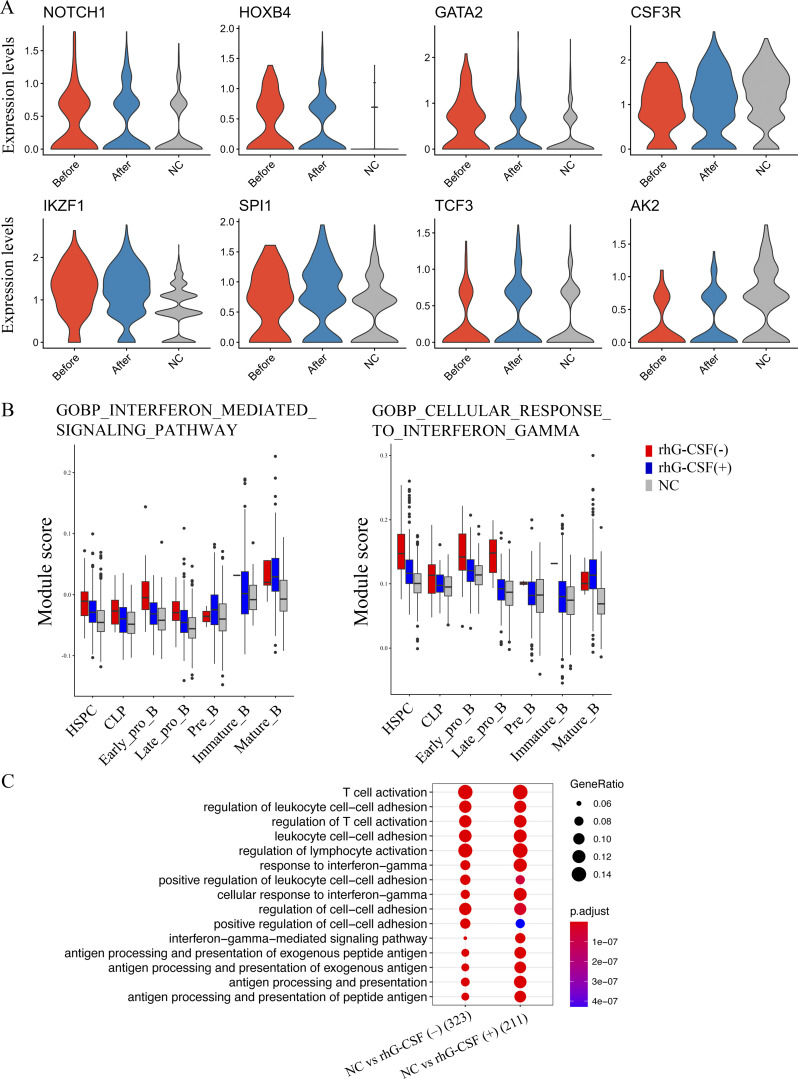
**Modulation of IFNγ-related signaling pathways following rhG-CSF administration. (A)** Gene expression of developmental markers and transcription factors in the HSPC fraction was compared across the three groups. **(B)** Gene expression of IFNγ-related signaling pathways in each B cell fraction was compared across the three groups. In the B cell fractions, including HSPC and CLP, IFNγ-related signaling pathways were significantly downregulated in the rhG-CSF group. **(C)** We assessed signaling alterations in the T cell fraction, and found that signaling pathways associated with T cell activation were upregulated both with and without rhG-CSF administration, compared with the NC group. CLP, common lymphoid progenitors; GOBP, Gene Ontology Biological Process; HSPC, hematopoietic stem and progenitor cell; NC, normal control.

Next, we evaluated differences in the HSPC fraction across the three groups to investigate the mechanisms underlying the progression of B cell differentiation and development in response to rhG-CSF administration (Fig. 3, A and B). The gene expression of major developmental markers and transcription factors in the HSPC fraction, as well as *AK2*, was comparable between the three groups (Fig. S2 A). Enrichment analysis of DEGs in the HSPC fraction demonstrated a predominant activation of nuclear factor κ-light-chain-enhancer of activated B cells (NF-κB) signaling and lymphocyte differentiation pathways without rhG-CSF administration (Fig. 3 C). Of the genes that were significantly upregulated without rhG-CSF administration compared with NC, 244 genes were significantly downregulated following rhG-CSF administration (Fig. 3 D). Using these genes, we calculated module scores at the single-cell level in Fig. 3 E, and performed gene set enrichment analysis specifically on the HSPC population. The results revealed enrichment of IFNγ-related signaling pathways and cytokine production, suggesting that rhG-CSF administration may suppress IFNγ-related gene sets in HSPCs, potentially contributing to the observed increase in B cell differentiation (Fig. 3 F and Fig. S2 B). Furthermore, a comparison of signaling alterations in T cells between the NC group and the groups with or without rhG-CSF administration showed marked activation of T cells in both with and without rhG-CSF administration (Fig. S2 C). *IFNG* gene expression was confined to T cells in all three groups (Fig. 3 G), with a notable decrease in the rhG-CSF(+) group (Fig. 3 H). This reduction in *IFNG* gene expression is consistent with the suppressed IFNγ pathways with rhG-CSF administration.

### Literature review

**Figure S3.**
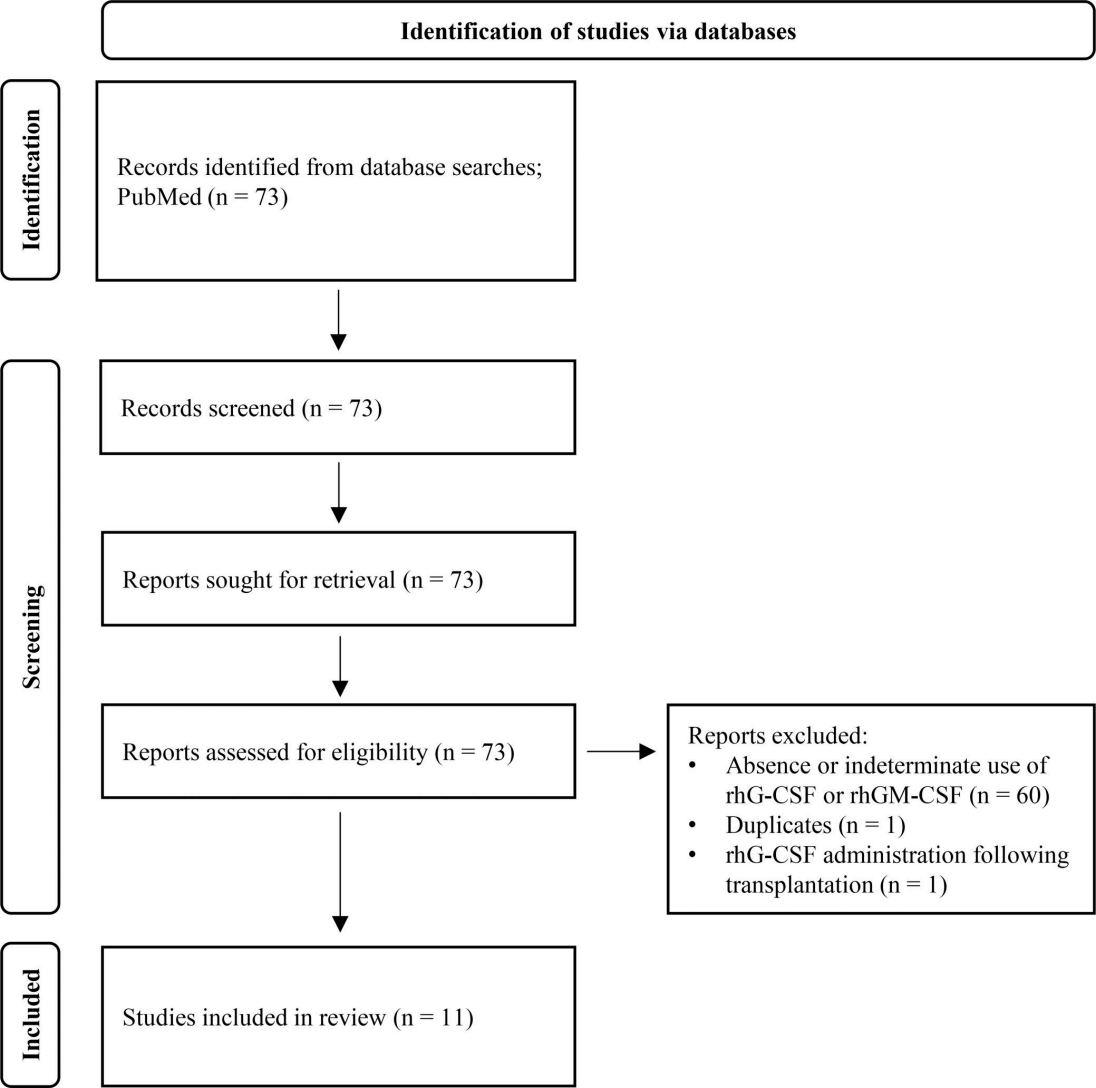
**PRISMA flow diagram for literature review on the significance of rhG-CSF/rhGM-CSF in patients with RD.** We reviewed 73 articles with the term reticular dysgenesis extracted from PubMed by February 2025, and identified 11 literature papers describing a total of 12 patients with RD who received rhG-CSF (*n* = 11) or rhGM-CSF (*n* = 1). RD, reticular dysgenesis; rhG-CSF, recombinant human granulocyte colony-stimulating factor; rhGM-CSF, recombinant human granulocyte–macrophage colony-stimulating factor.

We reviewed 73 articles with the term “reticular dysgenesis” extracted from PubMed by February 28, 2025. We identified 11 literatures describing a total of 12 patients with RD who received rhG-CSF (*n* = 11) or recombinant human granulocyte–macrophage colony-stimulating factor (rhGM-CSF; *n* = 1, patient #8) before their first allogeneic HSCT (Fig. S3), and these cases are summarized in Table 2. 10 out of 12 patients started treatment with rhG-CSF or rhGM-CSF within the first month after birth. Of these, two patients (patients #3 and #8) had increased neutrophil counts in response to rhG-CSF or rhGM-CSF administration. Patients #3 suffered from severe sepsis and pneumonia due to *Haemophilus influenzae* and *Pseudomonas aeruginosa* at 10 mo of age (12). At 13 mo of age following the RD diagnosis, he responded well to rhG-CSF administration with an increased neutrophil value; however, its use was discontinued because of Sweet’s disease, including acute febrile neutrophilic dermatosis with fever and the sudden onset of a rash. Patient #8, a 2-mo-old boy, was treated with rhGM-CSF, which increased the absolute neutrophil counts (13). In contrast, no published studies have discussed the effects of rhG-CSF or rhGM-CSF on B lymphocyte counts in patients with RD who were treated with rhG-CSF or rhGM-CSF.

**Table 2.** Literature review of patients with RD treated with rhG-CSF/GM-CSF before the first HSCT

No.	Sex	*AK2* variants	Age at initiation of rhG-CSF or GM-CSF	rhG-CSF or rhGM-CSF	Dose of rhG-CSF or rhGM-CSF (μg/kg/day)	Duration (days)	Response of neutrophils to rhG-CSF or rhGM-CSF	HSCT	Outcome	Cause of death	Ref
1	M	c.523C>G, p.Arg175Gly, and c.330+1G>C	16 days	rhG-CSF	10–20	13	No	No	Death	Sepsis	(14)
2	M	c.330+5G>A (homozygous)	1 day	rhG-CSF	ND	ND	No	Yes	Alive	-	(15)
3	M	c.622T>C, p.Ser208Pro (homozygous)	13 mo	rhG-CSF	2–3	7 mo^a^	Yes	Yes^b^	Death	SOS	(12)
4	M	c.524G>C, p.Arg175Pro (homozygous)	Within the first 3 mo	rhG-CSF	5	14	No	Yes	Alive	-	(16)
5	F	ND	Within the first 10 days	rhG-CSF	10	ND	No	No	Death	Sepsis	(17)
6	F	ND	1 mo	rhG-CSF	ND	ND	No	Yes^c^	Alive	-	(18)
7	M	ND	2 days	rhG-CSF	10	10	No	Yes^d^	Alive	-	(19)
8	M	ND	1 mo	rhGM-CSF	4–15	43	Yes	No	Death	Infections due to CMV and *Aspergillus fumigatus*	(13)
9	F	ND	1 day	rhG-CSF	4	14	No	Yes	Alive	-	(20, 21)
10	M	ND	21 days	rhG-CSF	5–30	40	No	Yes	Alive	-	(21)
11	ND	ND	16 days	rhG-CSF	10–20	ND	No	Yes	Alive	-	(22)
12	ND	ND	ND	rhG-CSF	10–100	ND	No	Yes	ND	-	(22)
UPN1	F	c.409C>T, p.Arg137Ter, and c.498+1G>A	11 days	rhG-CSF	5	129	Yes	Yes	Alive	-	This study
UPN2	F	c.308G>A, p.Arg103Gln, and c.409C>T, p.Arg137Ter	2 days	rhG-CSF	7.5–15	78	Yes	Yes	Alive	-	This study

CMV, cytomegalovirus; rhG-CSF, recombinant human granulocyte colony-stimulating factor; rhGM-CSF, recombinant human granulocyte–macrophage colony-stimulating factor; HSCT, hematopoietic stem cell transplantation; ND, no data; SOS, sinusoidal obstruction syndrome; UPN, unique patient number; MDS, myelodysplastic syndromes.

a Discontinued due to the development of Sweet’s syndrome.

b The second HSCT was performed because the patient developed neutropenia and complete recipient chimerism in the myeloid lineage at 6 mo after transplant.

c Two months after HSCT, neutrophils gradually decreased, leading to mixed chimerism, and a second HSCT was performed at 8 mo.

d The first HSCT was performed without a conditioning regimen, followed by a second transplant from the same donor.

## Discussion

Patients with RD are typically unresponsive to rhG-CSF administration due to intrinsic hematopoietic defects caused by AK2 deficiency, as defined in the diagnostic criteria (23). However, in our two cases of RD, short-term rhG-CSF administration before HSCT, especially in those requiring treatment for severe infections, might be beneficial in preventing serious bacterial infections and infection-related complications. Even slight improvements in neutrophil counts could have clinically meaningful implications for infection control in RD. Furthermore, this literature review identified two patients with RD who exhibited an increased neutrophil count following rhG-CSF or rhGM-CSF administration. The rhG-CSF or rhGM-CSF administration could be considered a supportive therapy for this disease during the pretransplantation period, especially when facing life-threatening bacterial infections. The widespread implementation of TREC/KREC NBS programs is estimated to increase the number of patients with RD who can be diagnosed at the early disease stages, for whom the use of rhG-CSF could be discussed.

In this study, we demonstrated differentiation and maturation of B cell fractions with rhG-CSF administration and downregulation of IFNγ-pathway related genes in HSPC fraction, suggesting a potential role of IFNγ signaling in this process. IFNγ is an essential cytokine in both immune and inflammatory responses, and is well established to inhibit B cell differentiation under certain conditions (24, 25). Elevated levels of *IFNG* and *CXCL9* have been identified in patients with HLH and its animal models, and IFNγ neutralization has been implicated in contributing to improved clinical outcomes (26). Similarly, in patients with AA, T cells expressing *IFNG* have been shown to significantly decrease after immunosuppressive therapy, which correlates with hematologic improvement (9). Dysregulated IFNγ signaling might underlie disrupted hematopoiesis in RD, indicating the IFNγ pathway as a potential therapeutic target, and future analyses of total bone marrow IFNγ levels would help clarify its role in this process. We cannot exclude the possibility that G-CSF–induced immunoregulatory effects mediated by monocytes or dendritic cells also contribute to the observed modulation of T cell IFNγ production (27). In addition, although certain drugs may theoretically influence cytokine signaling, we do not believe that other concomitant medications substantially impacted IFNγ levels in this case. Consistently, gene set enrichment analysis showed activation of NF-κB signaling pathways in the HSPC in the absence of rhG-CSF. Activation of NF-κB signaling supports B cell survival and differentiation, potentially counterbalancing the suppressive effects mediated by the IFNγ pathway (28). Further investigations are needed to elucidate the immunomodulatory effects of rhG-CSF and to clarify the molecular basis of hematopoietic regulation in RD.

Long-term administration of rhG-CSF, however, may require careful monitoring. In prior cases of patients with RD who underwent HSCT, mixed chimerism was observed, and two patients developed myelodysplastic syndromes (MDS) after prolonged rhG-CSF treatment for 2 and 4 years, respectively. These observations suggest that extended rhG-CSF therapy in RD may be associated with an increased risk of MDS, underscoring the importance of ongoing monitoring and risk assessment during treatment (29).

**Figure S4.**
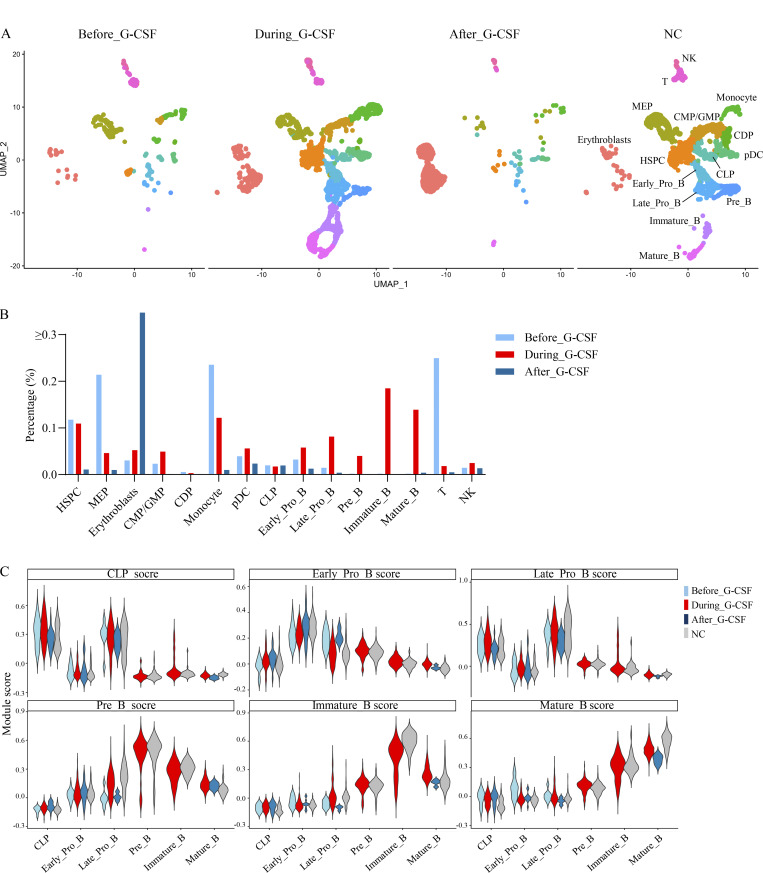
**Alterations in lineage-specific gene expression signatures across HSPC and B cell differentiation before, during, and after rhG-CSF administration. (A)** Uniform Manifold Approximation and Projection embedding of hematopoietic cells sampled before, during, and after rhG-CSF administration and from NCs. Each dot represents a single cell. **(B)** Relative proportions of each cell population under before, during, and after rhG-CSF administration. **(C)** Violin plots showing gene expression signature scores for B cell lineage stages across conditions (CLP, early pro-B, late pro-B, pre-B, immature B, mature B). CLP, common lymphoid progenitors; CMP, common myeloid progenitors; GMP, granulocyte–macrophage progenitors; HSPC, hematopoietic stem and progenitor cells; MEP, megakaryocyte–erythroid progenitor; NK, natural killer cell; pDC, plasmacytoid dendritic cell; rhG-CSF, recombinant human granulocyte colony-stimulating factor.

This study has several limitations. First, our findings are constrained by the small sample size of only two patients with RD, which limits the generalizability of our results and warrants caution in interpreting changes in specific cell populations, such as the observed decrease in erythroblast frequency in the rhG-CSF(+) group. Furthermore, because BM samples from patients with RD are profoundly depleted of hematopoietic cells across multiple lineages, TP1 (before rhG-CSF initiation) and TP4 (after rhG-CSF withdrawal) could not be analyzed as fully independent conditions without compromising the interpretability of the data (Fig. S4). RD is an extremely rare disorder, and although these patients provide valuable insights, larger studies are necessary to validate our findings. Second, the exact mechanisms behind the responses of neutrophils and B lymphocytes to rhG-CSF, as well as the selective B cell differentiation following the downregulation in IFNγ-related signaling pathways, remain unclear. Further studies are needed to explore the mechanisms of rhG-CSF in RD and to refine treatment strategies for this rare immunodeficiency disorder.

In conclusion, we demonstrated that rhG-CSF administration before HSCT may be clinically beneficial for patients with RD by moderately increasing neutrophil counts and contributing to infection management. Furthermore, rhG-CSF administration was associated with decreased expression of *IFNG* and its related pathways in the HSPC fraction, and potentially promoted B cell differentiation in patients with RD.

## Materials and methods

### Patients

BMNC samples were obtained from two female patients with RD (with and without rhG-CSF treatment), as well as from three pediatric healthy individuals (NCs). Written informed consent was obtained from the patients’ parents before sample collection. This study was performed in line with the principles of the Declaration of Helsinki. Approval was granted by the Ethics Committee of the Nagoya University Graduate School of Medicine (approval number 2015-0035).

### Whole-exome sequencing

Genomic DNA was extracted using QIAamp DNA Blood Mini Kit (QIAGEN) from PB cells. The extracted DNA was then captured using SureSelect Human All Exon 50 M, V6 Kits (Agilent Technologies). Sequencing of the captured DNA was carried out on a NovaSeq 6000 next-generation sequencer (Illumina), with a 150 × 2 paired-end option, and germline variants were identified using the Genomon whole-exome sequencing pipeline as previously described (30).

### scRNA-seq library preparation and sequencing

Single-cell 3′ RNA sequencing analysis (scRNA-seq; 10x Genomics) was performed in two patients with RD (UPN1 and UPN2) under conditions with and without rhG-CSF administration. As NCs, we performed scRNA-seq using preserved BMNC from three pediatric NC samples. FACSAria Fusion (BioLegend) with anti-human CD34 antibody (BioLegend) and anti-human Lineage Cocktail (BioLegend) was used to sort CD34-positive and lineage-negative (CD34^+^Lin^−^) fractions from BMNC. For single-cell gene expression analysis, in RD patient samples, the enriched CD34^+^Lin^−^ fraction was mixed with bulk BMNCs at a 1:5 ratio. In the NC samples, CD34^+^ cells were combined with BMNCs at an ∼1:1 ratio. The Chromium Next GEM Single-Cell 3′ Reagent kit v3.1 (10× Genomics) was used to generate cDNA libraries following the manufacturer’s instructions. Libraries were sequenced using an Illumina NovaSeq 6000 (Macrogen Japan Corp.).

### Data analysis of scRNA-seq

The alignment, barcode processing, and unique molecular identifiers of the sequencing data were counted using 10× Cell Ranger (version 6.1.2) (10× Genomics). The downstream work was performed using the R package Seurat (version 4.0.6) (31). Quality control was performed by filtering mitochondrial expression (*n* < 10%) and unique transcripts (200 <  *n* < 9,000). scRNA-seq data from three pediatric NC samples were used to define genes that are significantly differentially expressed in each hematopoietic cell population. DEGs for each cluster of NC samples were extracted based on an adjusted P value (P < 0.05), and the top 200 genes were used for downstream analysis. Module scores for each cell population were calculated with the AddModuleScore function based on the significantly DEGs or B cell differentiation–related pathways registered in Gene Ontology (32). DEGs were identified using the Seurat FindAllMarkers function for cluster-specific marker discovery, and the FindMarkers function for comparing HSPC conditions before and after rhG-CSF administration. The Wilcoxon rank-sum test was used to calculate the statistical significance. We analyzed the enrichment of Gene Ontology using the R clusterProfiler package (version 4.10.1) (33) based on the DEGs identified by gene expression analysis.

### Literature review

A systematic literature review was conducted based on the Preferred Reporting Items for Systematic Reviews and Meta-Analyses guidelines using a combination of controlled vocabulary, when applicable, and keywords. Literature in the PubMed database (https://pubmed.ncbi.nlm.nih.gov) was searched on February 28, 2025, and articles published until February 2025 were included. The search terms were reticular dysgenesis [All Fields] AND (“0000”[PDAT]: “2025/2/28”[PDAT]).

### Online supplemental material

The online supplemental material provides additional methodological details and extended analyses supporting the findings of this study. Fig. S1 shows the expression of canonical marker genes across annotated hematopoietic cell populations, confirming cell-type assignments. Fig. S2 presents alterations in IFNγ-related signaling pathways following rhG-CSF administration across HSPC, B cell, and T cell fractions. Fig. S3 provides a PRISMA flow diagram summarizing the literature review on the clinical significance of rhG-CSF/rhGM-CSF in RD. Fig. S4 illustrates dynamic changes in lineage-specific gene expression signatures and cellular composition before, during, and after rhG-CSF administration.

## Data Availability

The datasets analyzed during the current study are available from the corresponding author on reasonable request.
